# Measurement of the Infection and Dissemination of Bluetongue Virus in *Culicoides* Biting Midges Using a Semi-Quantitative RT-PCR Assay and Isolation of Infectious Virus

**DOI:** 10.1371/journal.pone.0070800

**Published:** 2013-08-05

**Authors:** Eva Veronesi, Frank Antony, Simon Gubbins, Nick Golding, Alison Blackwell, Peter PC. Mertens, Joe Brownlie, Karin E. Darpel, Philip S. Mellor, Simon Carpenter

**Affiliations:** 1 Vector-borne Viral Diseases Programme, The Pirbright Institute, Pirbright, Surrey, United Kingdom; 2 Advanced Pest Solutions Ltd., Dundee, United Kingdom; 3 Royal Veterinary College, Hatfield, Herts, United Kingdom; 4 University of Surrey, Guildford, Surrey, United Kingdom; University of Georgia, United States of America

## Abstract

**Background:**

*Culicoides* biting midges (Diptera: Ceratopogonidae) are the biological vectors of globally significant arboviruses of livestock including bluetongue virus (BTV), African horse sickness virus (AHSV) and the recently emerging Schmallenberg virus (SBV). From 2006–2009 outbreaks of BTV in northern Europe inflicted major disruption and economic losses to farmers and several attempts were made to implicate Palaearctic *Culicoides* species as vectors. Results from these studies were difficult to interpret as they used semi-quantitative RT-PCR (sqPCR) assays as the major diagnostic tool, a technique that had not been validated for use in this role. In this study we validate the use of these assays by carrying out time-series detection of BTV RNA in two colony species of *Culicoides* and compare the results with the more traditional isolation of infectious BTV on cell culture.

**Methodology/Principal Findings:**

A BTV serotype 1 strain mixed with horse blood was fed to several hundred individuals of *Culicoides sonorensis* (Wirth & Jones) and *C. nubeculosus* (Mg.) using a membrane-based assay and replete individuals were then incubated at 25°C. At daily intervals 25 *Culicoides* of each species were removed from incubation, homogenised and BTV quantified in each individual using sqPCR (C_q_ values) and virus isolation on a KC-*C. sonorensis* embryonic cell line, followed by antigen enzyme-linked immunosorbent assay (ELISA). In addition, comparisons were also drawn between the results obtained with whole *C. sonorensis* and with individually dissected individuals to determine the level of BTV dissemination.

**Conclusions/Significance:**

C_q_ values generated from time-series infection experiments in both *C. sonorensis* and *C. nubeculosus* confirmed previous studies that relied upon the isolation and detection of infectious BTV. Implications on the testing of field-collected *Culicoides* as potential virus vectors by PCR assays and the use of such assays as front-line tools for use in diagnostic laboratories in this role are discussed.

## Introduction

In 2006, bluetongue virus (BTV) emerged in northern Europe for the first time in recorded history, inflicting severe economic damage on the livestock sector in this region [1], [2]. The transmission of BTV occurs primarily by a biological, propagative cycle in competent *Culicoides* biting midges [3], [4]. Risk of BTV emergence in new regions is hence, in part, assessed by the seasonal and/or spatial presence or absence of adult *Culicoides* and the strength of evidence implicating the species present as a transmission threat. Prior to the incursion of BTV into northern Europe, clear evidence existed that some *Culicoides* species present in this region were capable of transmitting at least some strains of BTV [1]. The most likely potential vectors were the *C. obsoletus* group (more correctly, those species placed within the Avaritia subgenus in this region: *Culicoides obsoletus* Meigen, *Culicoides scoticus* Downes & Kettle, *Culicoides dewulfi* Goetghebuer and *Culicoides chiopterus* Meigen). Some of these species had already been implicated in BTV transmission in southern Europe by one or more of: (a) isolation of BTV from pools of *Culicoides* caught at light [5]–[9]; (b) congruence of their distribution or seasonality with BTV outbreak sites [10]–[12]; or (c) the ability of populations in the UK to replicate BTV to high titres in the laboratory [13], [14]. A key issue that arises in attempts to implicate *Culicoides* as biological vectors is the requirement to show an arbovirus is not only present and detectable in a putative vector, but also that it has replicated and disseminated within at least a proportion of the population. Infection of the salivary glands in vector *Culicoides* and subsequent presence of the arbovirus in saliva are required for transmission to occur [4].

Following the incursion of BTV into northern Europe, a series of studies were conducted which attempted to implicate *Culicoides* collected at outbreak sites in transmission of the virus [15]–[17]. These studies utilised real-time reverse transcription polymerase chain reaction assays (rtRT-PCR) to detect viral RNA within pools of *Culicoides* without subsequent virus isolation. This was largely because rtRT-PCR was readily available in national reference laboratories for BTV that were already processing large numbers of ruminant-derived samples. A virtual absence of data concerning BTV RNA quantity, however, meant that results were difficult to interpret. ‘Positive’ findings of detected viral RNA may merely have represented inactivated BTV that had persisted in individual *Culicoides* following an infected blood meal, or a sub-transmissible infection such as commonly occurs in this genus [4]. Subsequent to these early studies, the inclusion of cycle threshold (C_q_) values as a semi-quantitative indication of RNA concentration has become more common [18], [19]. Uncertainty remains, however, in interpretation of semi-quantitative or sqPCR data from pools of *Culicoides* and in the relationship between C_q_ values representing quantity of viral RNA and the quantity of infectious virus as a means of defining transmissible infections.

In this study we compare C_q_ values generated by sqPCR with detection of infectious virus in two colony populations of *Culicoides*, (*C. nubeculosus* and *C. sonorensis*) that were fed artificially through membranes on blood/virus suspensions. *C*. *nubeculosus* is distributed across the Palaearctic region and has already been found to be only poorly competent for BTV in a series of laboratory experiments conducted primarily using the single colony line also used in the present study [13]. In contrast, *C*. *sonorensis* is a major vector of BTV in the USA [20] and the colony line used has been shown to be competent for many BTV types and strains [21], [22]. By comparing and contrasting the replication of BTV over time in these two colony lines of *Culicoides* species, as measured using the rtRT-PCR and traditional virus isolation techniques, we assess methods currently used to identify field-collected *Culicoides* as vectors of BTV and other arbovirus species such as the recently emerging Schmallenberg virus [23].

## Materials and Methods

Batches of approximately three to four hundred 2–3 day old adult *C. sonorensis* and *C. nubeculosus* were allowed to feed on a defibrinated horse-blood (TCS Biosciences, UK)/virus suspension via the Hemotek system (Discovery Workshops, Accrington, UK) using a Parafilm® membrane (Cole-Parmer, Hanwell, UK). The *C. sonorensis* were from the PIRB-s-3 strain, originally derived from the USA SONORA line [24] and acquired by the laboratory in the 1970s, while the *C. nubeculosus* originated from the UK and have been maintained at The Pirbright Institute for a similar time period [25]. The virus used during the trial was derived from the ‘western topotype’ strain of BTV-1 that was originally isolated in KC cells (an embryonic cell line derived from *C. sonorensis* and originally created in the USA [26]) from an outbreak in Gibraltar (for further details see http://www.reoviridae.org/dsRNA_virus_proteins/ReoID/btv-1.htm#GIB2007/01). The virus used had a C_q_ of 16.74 prior to combining with blood and contained 6.5 Log_10_ TCID_50_/ml infectious BTV prior to mixing 1∶1 with horse blood.

Fully engorged, blood-fed individuals were selected under light CO_2_ anaesthesia (defined as being sufficient to immobilise the *Culicoides* but without constant and prolonged exposure) and placed in clean waxed cardboard pill boxes (Watkins and Doncaster, Stainton, UK) with fine mesh tops. Boxes were then placed in incubators at a constant temperature of 25°C with a pad of cotton wool soaked in 5% sucrose provided on the mesh as an energy source; this was replaced daily. Immediately post-feeding and subsequently at 24 hour intervals for 11 days, 25 individuals of each species were removed from incubation, anaesthetised using CO_2_, and placed in racked 96 sample polypropylene tubes (Qiagen, Crawley, UK) containing 200 µl of Glasgow Minimum Essential Medium (GMEM: Invitrogen, Paisley, UK). These samples were immediately homogenised using 3 mm stainless-steel ball-bearings (Dejay Distribution Ltd, UK) in a Tissuelyser™ (Qiagen, Crawley, UK) by shaking at 25 hz for one minute [27]. Tubes were centrifuged briefly at 1000 rpm and their sealing caps were removed to allow addition of a further 800 µl of GMEM with antibiotics (1000 IU/ml Penicillin/Streptomycin; 4 µg/ml Amphotericin B), the stainless steel ball-bearing was then removed, followed by an additional centrifugation for 3 mins at 3000 rpm. Processing of samples for BTV RNA and infectious virus occurred up to a maximum of three days following this treatment, during which time samples were stored at 4°C. Samples were titrated to a maximum ≥4.5 log_10_ TCID_50_ for *C. sonorensis* and ≥2.5 log_10_ TCID_50_ for *C. nubeculosus*. These titration limits allowed correlation between levels of infectious virus and BTV RNA to be investigated in the case of *C. sonorensis* while the maximum of 2.5 log_10_ TCID_50_ for *C. nubeculosus* represented a previously defined cut-off value for transmissible infections [28].

### Dissemination of BTV in *C. sonorensis*

#### Intrathoracic Inoculation (IT)

Approximately 100 *C. sonorensis* were inoculated with the BTV-1 strain parental to that described in the previous section (KC_2_; C_q_  = 14.88; 7 Log_10_TCID_50_) using pulled glass capillary needles (Narishige, Japan) and a microinjector with a manual syringe driver (Sutter Instruments, USA). Inoculation was carried out under light CO_2_ anaesthesia and surviving midges were transferred to pill boxes and incubated for 11 days at 25°C. *Culicoides sonorensis* were then immobilized using CO_2_ and fixed to a piece of masking tape with their ventral surface exposed. A drop of pillocarpine (parasympathomimetic alkaloid: Sigma Aldrich, UK) solution was then applied to the ventral surface of each *C. sonorensis* and saliva collected into a 1 µl microcapillary glass tube containing 10% foetal bovine serum (FBS) supplemented Glasgow’s media (GMEM). The collected media was then expelled into individual Eppendorf tubes containing 0.5 ml of Schneider’s *Drosophila* Media (Gibco™) containing 10% FBS. Individuals were then decapitated using a needle (Monoject™ hypodermic needle, 18 g × 1.5: Covidien, USA) and the heads were ground, in 3–4 µl of GMEM supplemented with 10% foetal bovine serum, between washed glass cover-slips. The homogenate was collected into a 1.5 ml Eppendorf containing 1 ml of GMEM with antibiotics (1000 IU/ml Penicillin/Streptomycin; 4 µg/ml Amphotericin B). Finally, the thorax and abdomen were homogenised together in a sterile Eppendorf tube and processed in the same manner as the whole insect samples described in the previous section. All homogenates were subsequently tested using both sqPCR (in duplicate) and by KC-cell titration followed by detection of BTV antigens by ELISA to a maximum detectable titre of ≥5.5 log_10_ TCID_50_.

#### Membrane feeding

*Culicoides sonorensis* were fed as previously described, with BTV-1w GIB2007/1 (KC2 passage) and incubated at 25°C for seven days. Saliva was collected from sixty surviving *C. sonorensis* as for IT inoculation and then dissected under CO_2_ anaesthesia using fine needles to divide bodies into head, thorax and abdominal sections. The heads were ground as for IT inoculation, while the thorax and abdomen sections were homogenised as for whole insects. All samples were subsequently tested using the sqPCR followed by KC-cell titration and antigen ELISA to a maximum detectable titre of ≥6 log_10_ TCID_50_.

### Effect of Pooling Upon Detection of BTV RNA in *Culicoides*

*Culicoides sonorensis* were infected using IT inoculation as described above, using the KC3 passage of BTV-1w GIB2007/1. The insects were then incubated for 7 days at 25°C before homogenization in pools, with a variable number of uninfected individuals, in 300 µl of GMEM for 1 minute at 25 Hz using a 3 mm stainless-steel ball bearing. The numbers of infected: uninfected *C. sonorensis* tested were: 1∶2, 1∶20, 1∶50 and 1∶100 with three replicates of each pool. The tubes were then each filled with a further 1.7 ml of GMEM with antibiotics and filtered (0.80 µm pore: Sartorius, Epsom, UK) using a 2 ml disposable syringe and the resulting supernatant stored as described above for up to three days at 4°C.

### Detection of BTV

#### Real-time RT-PCR

Nucleic acid extraction was carried out using a Universal Biorobot (Qiagen, Crawley, UK) in a 96-well format using a QIAamp® All Nucleic Acid MDx Kit (Qiagen, Crawley, UK). Forty microlitres of reconstituted protease were added to each well, followed by 50 µl of the sample, 190 µl of nuclease free water and 360 µl of lysis/binding buffer (Roche Diagnostics, Lewes, UK). SqPCR reactions were carried out according to a standardised assay [29]. Each 19 µl of reaction mastermix contained the following: 12.5 µl 2× reaction buffer mix, 1 µl of 20 pmol solution of each primer, 0.5 µl of 2.5 pmol each probe, 0.5 µl Mg_2_SO_4_, 0.5 µl 1∶10 diluted ROX reference dye and 0.5 µl of the Superscript III/Platinum *Taq* enzyme mix (Invitrogen, Paisley, UK). Six microlitres of denatured RNA was added to the reaction mix and amplification was carried out in an Mx3005P PCR machine (Agilent Technologies, Stockport, UK). Fluorescence was measured at the end of the 60°C annealing/extension step. Cycle threshold values for each sample were determined from the point at which threshold fluorescence was breached.

#### Indirect antigen sandwich ELISA

Quantification of infectious virus titre were carried out by application of serially diluted homogenised insect samples to KC monolayers in 96-well plates and then processed using a modified version of an antigen ELISA [30]. Plates were covered and incubated for seven days at 25°C, then 50 µl of supernatant from each well was transferred to an ELISA (Nunc) plate pre-coated (O/N at 4°C) with 50 µl/well of rabbit antibody raised against the ‘western topotype’ reference strain of BTV-1 (RSArrrr/01) (http://www.reoviridae.org/dsRNA_virus_proteins/ReoID/btv-1.htm#RSArrrr/01) at a dilution of 1∶2000 in coating buffer. Guinea pig anti BTV-1 antiserum was then diluted 1∶2000 in blocking buffer (Tween-20 and 5% non-fat dried milk in PBST and 50 µl added to each well). Polyclonal rabbit anti-guinea pig immunoglobulins conjugated to HPR (DAKO Ltd, UK; 1∶1000 dilution in 5% marvel blocking buffer) was then added and plates incubated for an additional 45 minutes at 37°C. Subsequently, each plate was washed three times with PBS and 50 µl/well of substrate added (0.4 mg/ml o-Phenylenediamine dihydrochloride (Sigma, UK) and 0.05% hydrogen peroxidise solution). Plates were then incubated at room temperature for 10 minutes without covering. Following this, 1 M sulphuric acid was used to halt the reaction and plates were read with a 490 nm filter using an arbitrary cut off of <0.20 optical density above negative controls. Virus titres were calculated using the method of Spearman and Karber [31], with a range of detectable titre from 0.5 to 4.5 log_10_TCID_50_ in 0.25 log_10_TCID_50_ increments (*C. sonorensis*) or from 0.5 to 2.5 log_10_TCID_50_ in 0.25 log_10_TCID_50_ increments (*C. nubeculosus*).

### Statistical Methods

For *Culicoides* processed as ‘whole insects’ change over time in the proportion of individuals with infectious virus was assessed using a generalised linear model (GLM) assuming binomial errors and a logit link function with days post infection as a factor. Wilcoxon rank-sum tests were used to compare C_q_ values in *Culicoides* with infectious virus (>0.20 optical density on ELISA) and not detected on each day of sampling. For *Culicoides* which were membrane-fed and then dissected, C_q_ values were analysed using linear mixed models with body part (abdomen, thorax, head or saliva) and infection status (BTV detected or not detected) as fixed effects and individual as a random effect. Observations with no C_q_ value were right-censored arbitrarily at C_q_  = 50 to allow them to be included in the analysis. Viral titres for these midges were analysed in the same way, except that observations with a titre ≤0.5 log_10_ TCID50 were left-censored. For *Culicoides* which were intrathoracically inoculated and then dissected, titres and C_q_ values were analysed using linear mixed models with body part (abdomen/thorax, head or saliva) as a fixed effect and individual as a random effect.

## Results

### Infection of *Culicoides* by Artificial Feeding and Virus Replication Profile

From days 0 to 11, a total of 300 *C. sonorensis* were processed of which 162 individuals were positive for BTV RNA by sqPCR (mean C_q_: 31.63; range 17.96 to 44.59). Infectious virus was detected in 104 homogenates (median log_10_ TCID_50_: 2.5; range: ≤0.5 to ≥4.5), all of which contained detectable BTV RNA (mean C_q_: 29.49; range: 17.96 to 40.71). Infectious BTV was not detected in a further 58 homogenates that contained detectable BTV RNA (mean C_q_: 35.45; range: 24.96 to 44.59). Of the 300 *C. nubeculosus* that were tested, 93 homogenates contained detectable BTV RNA (mean C_q_: 32.94; range: 20.76 to 44.43) of which 71 homogenates contained detectable infectious virus of >0.5 log_10_ TCID_50_. No infectious BTV was detected in 22 *C. nubeculosus* containing BTV RNA (mean C_q_: 37.10; range: 32.87 to 44.43).

**Figure 1:**
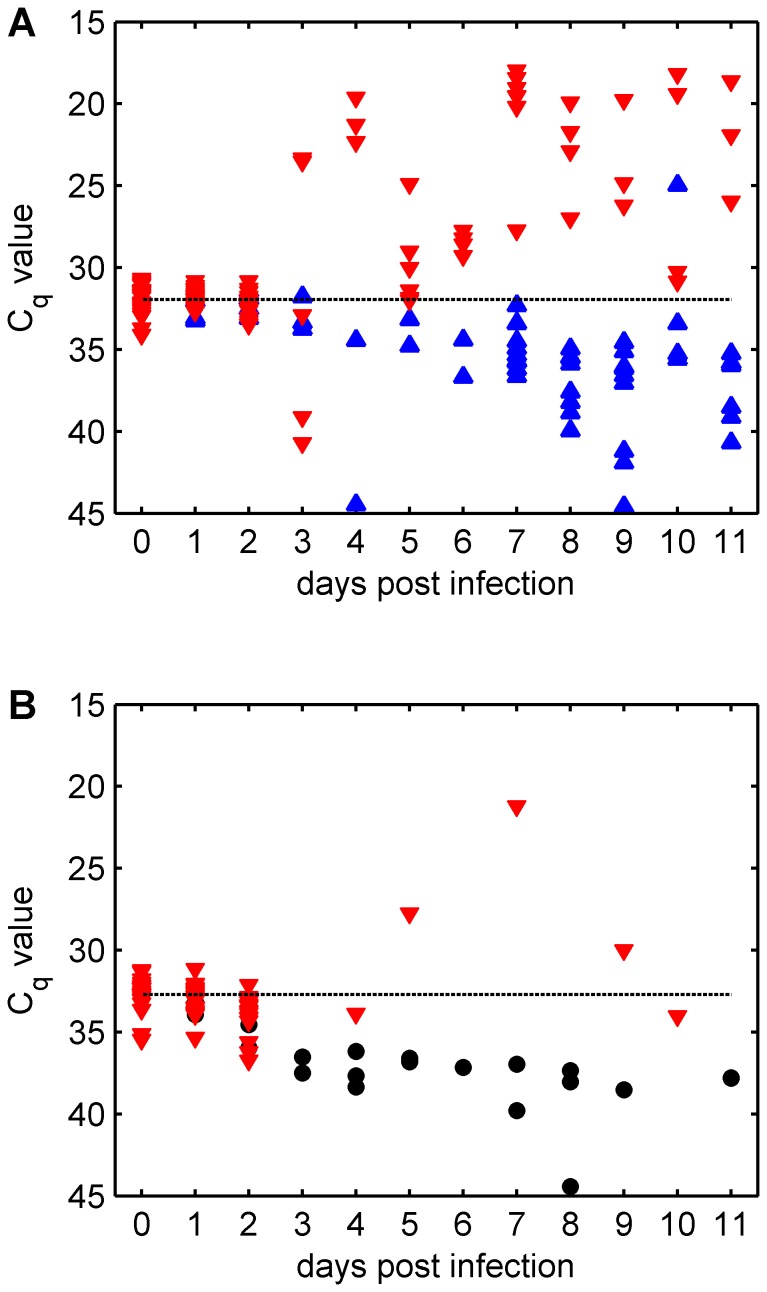
Changes over time in detection of total BTV RNA (as measured by C_q_ value). (A) *Culicoides sonorensis* and (B) *C. nubeculosus* were fed upon a BTV-1 strain via a membrane based system (note the inverted scales for the y-axes). In each figure the dashed line indicates the mean C_q_ value for *Culicoides* sampled on day 0. For *C. sonorensis* the symbols indicate whether (red down-triangles) or not (blue up-triangles) infectious BTV was isolated from the individual. For *C. nubeculosus* the symbols indicate whether infectious BTV was detected (red down-triangles) or the individual was not tested for infectious BTV (black circles).

**Figure 2:**
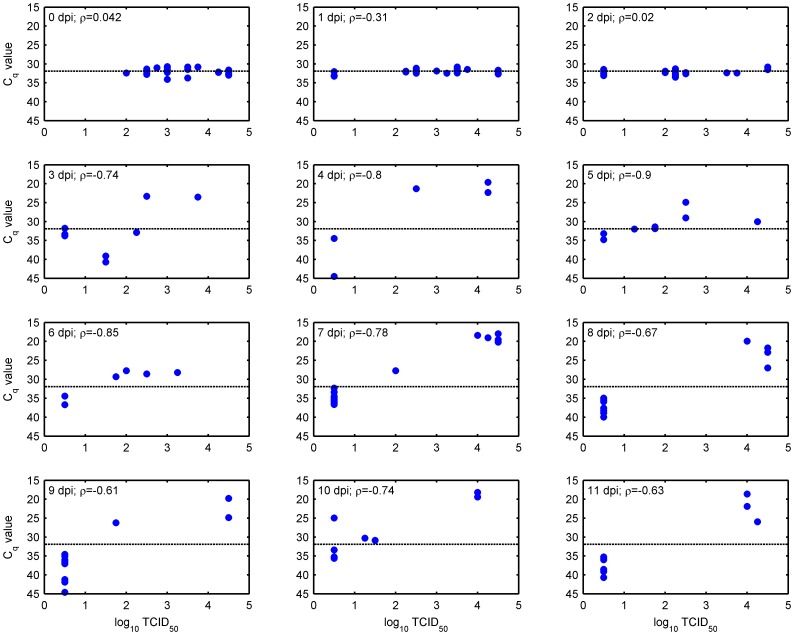
Relationship between viral titre (log_10_ TCID_50_) and viral RNA quantity (estimated using C_q_ values generated from sqPCR) in membrane-fed *C. sonorensis*. Results are shown for midges tested at different days post infection (dpi). Spearman’s correlation coefficient (ρ) was computed for each time point to compare titres and C_q_ values. All correlations were significantly (P<0.0.01) different from zero for 3–11 days post infection. In each figure the dashed line indicates the mean C_q_ value for individuals sampled on day 0.

The viral RNA load in the initial blood-meal recorded at day 0 differed significantly (*W*  = 485; P  = 0.003) between species with C_q_ values for *C. sonorensis* (mean: 31.94; range 30.69 to 34.09) being lower than those for *C. nubeculosus* (mean: 32.72; range: 31.23 to 35.47) as a consequence of body size. In *C. sonorensis*, infectious BTV was found in all day 0 individuals (median log_10_ TCID_50_: 3; range 2 to ≥4.5). The proportion of *C. sonorensis* in which infectious BTV was detected differed significantly (P<0.001) between samples on days 0 to 2 post infection (when blood containing virus was still present in the insects) and those sampled on days 3 to 11 post infection (following clearance of the blood meal). Significant differences in infection rate with BTV were not found, however, amongst samples within these time periods (Figures 1A and 2). The relationship between C_q_ values and quantity of infectious BTV was initially poorly correlated (Spearman’s ρ was not significantly different from zero (P>0.1)), but became significantly (P<0.001) negatively correlated from day 3 post infection following clearance of the original blood-meal (Figure 2).

Considering only those *C*. *sonorensis* tested three or more days post-infection, C_q_ values in which infectious virus was detected (mean C_q_: 25.28, range: 17.96 to 40.71) were significantly lower than those for individuals in which infectious virus was not detected (mean C_q_: 36.10, range: 24.96 to 44.59) (W = 106, P<0.001) (Figure 2). Furthermore, for *C. sonorensis* sampled between day 3 and day 11 post infection, 35 (out of 39) individuals for which infectious virus was detected had a C_q_ value below the mean recorded on day 0 (Figure 1A). By contrast, only 2 (out of 186) *C. sonorensis* in which infectious BTV was not detected had a C_q_ value below the mean on day 0 (Figure 1A). Vector competence (the proportion of individuals possessing fully disseminated infections) in *C. sonorensis* was estimated to be 16.4% (95% confidence interval (CI): 11.9–22.0%) based on the proportion of individuals with a C_q_ value below the mean on day 0 (37 out of 225) compared with 17.3% (95% CI: 12.6–22.9%) based on the proportion of midges in which infectious virus was additionally detected (39 out of 225). This difference was not statistically significant (χ^2^ = 0.06, df = 1, P = 0.80). In *C. nubeculosus* only four insects (out of 225) sampled three or more days post infection had C_q_ values below the mean on day 0 (Figure 1B). This implies a vector competence of 1.8% (95% CI: 0.5–4.4%), which is significantly lower than that recorded for *C. sonorensis* (χ^2^ = 29.2, df = 1, P<0.001).

### Dissemination of BTV in *C. sonorensis*

Survival rates of intrathoracically inoculated *C. sonorensis* were low, with approximately 5% of individuals surviving the 11 day incubation period (previously, survival rates have commonly exceeded 50% of individuals inoculated; Mellor pers. comm.). All five surviving individuals possessed fully disseminated infections that could be detected by sqPCR and by virus isolation. The largest quantity of viral RNA was found in the abdomen/thorax (mean C_q_: 18.17) followed by the head (mean C_q_: 21.99) and then the saliva (mean C_q_: 29.93). These differences were statistically significant (P<0.001). The quantity of infectious BTV was correlated with the C_q_ values, with the greatest quantity in the abdomen (median log_10_ TCID_50_: ≥5.5) followed by the head (median log_10_ TCID_50_: 4.5) and the saliva (median log_10_ TCID_50_: 3.13).

**Figure 3:**
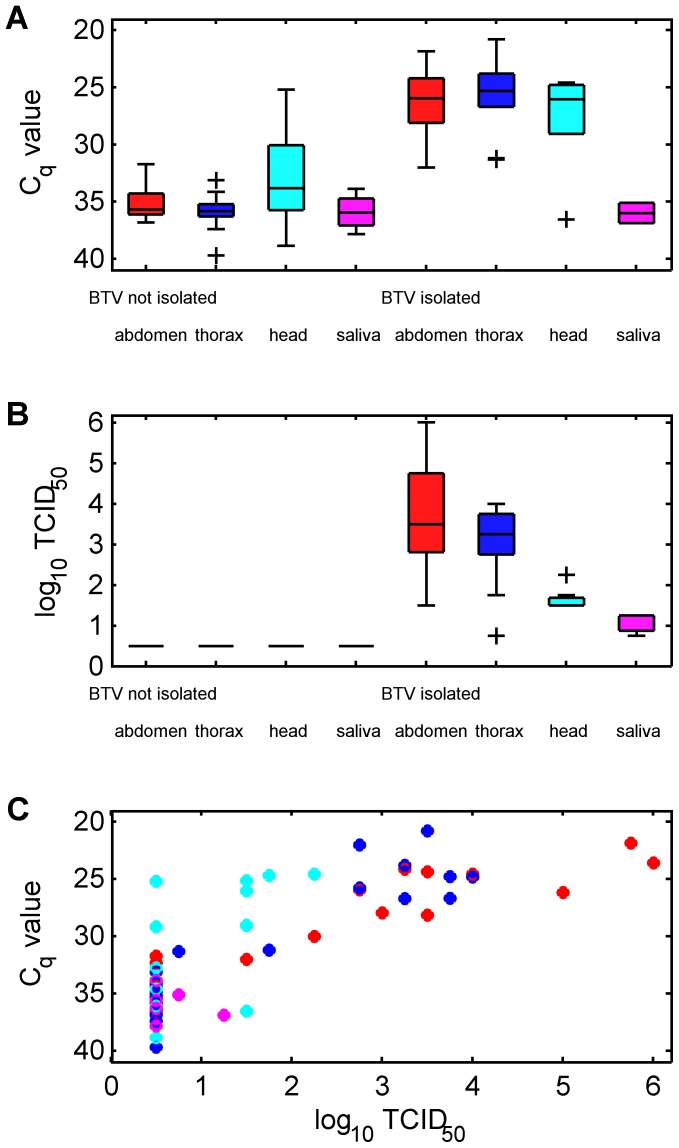
Measurement of BTV RNA and infectious virus in different body compartments for *C. sonorensis* incubated for 7 days at 25°C following feeding upon a BTV-1 strain via a membrane-based system. BTV RNA quantity is recorded by (A) C_q_ values and (B) viral titres (log_10_ TCID_50_). Insects are divided into those for which infectious virus was detected and those for which it was not. Box and whisker plots show the median (line), interquartile range (box), 1.5 times the interquartile range (whiskers) and any outliers (crosses) for C_q_ values in each body compartment. The relationship between viral titres and C_q_ values in each body compartment is shown in (C). Colours indicate body compartment: abdomen (red); thorax (blue); head (cyan); and saliva (magenta).

Eighteen (of 60) *C. sonorensis* that were membrane fed with BTV-1 generated duplicate samples containing BTV RNA in the separated abdomens at day seven post-infection. Eleven of these samples tested positive for infectious BTV (median log_10_ TCID_50_: 3.38; range: 1.5 to ≥6). Six *C. sonorensis* contained viral RNA in the head, thorax and abdomen and tested positive for infectious BTV across all body parts. The greatest quantities of BTV RNA and infectious virus were found in the abdomen (mean C_q_: 24.9; range: 21.68 to 28.94; median log_10_ TCID_50_: 4; range: 3 to ≥6) and the thorax (mean C_q_: 23.36; range: 20.57 to 25.47; median log_10_ TCID_50_: 3.38; range: 2.75 to 3.75), while similar levels of RNA but less infectious virus was found in the head (mean C_q_: 25.03; range: 24.51 to 28.29; median log_10_ TCID_50_: 1.5; range: 1.5 to 2.25). Viral titres for midges in which infectious BTV was detected differed significantly (P<0.006) between all body compartments, with the highest titres in the abdomen, followed by the thorax then the head, with the lowest titres in saliva (Figure 3B). Viral titres and C_q_ values were significantly (P<0.008) negatively correlated (Spearman’s ρ<−0.40) for all body compartments. Comparing the results for *C. sonorensis* processed as whole insects and those for the dissected individuals indicates that the proportion with fully-disseminated infections does not differ significantly from the proportion of insects with a C_q_ value below the mean on day 0 (χ^2^ = 3.26, df = 1, P = 0.07). In addition, the whole-body C_q_ values for *C. sonorensis* positive for BTV RNA sampled between days 3 and 11 post infection were comparable to those for the dissected abdomen (W = 374, P = 0.17) or thorax (W = 373, P = 0.18) of insects with a fully disseminated infection held for 7 days post-infection.

### Effect of Pooling Upon Detection of BTV in *Culicoides*

All pools processed contained detectable BTV RNA and infectious virus (Table 1). The C_q_ values recorded for pools were similar irrespective of the number of uninfected *C. sonorensis* included, although more variation in infectious titre was present in pools containing 49 and 99 uninfected *C. sonorensis*.

**Table 1:** Detection of single fully infected *C. sonorensis* among pools of uninfected individuals processed using an optimised Tissuelyser® assay (n  = 3 in all cases).

Number of fully infected*C. sonorensis* in pool (uninfected)	Mean C_q_ (range)	Median infectious bluetonguevirus titre log_10_ TCID_50_ (range)
1 (1)	23.98 (23.65–24.17)	2.75 (2.75–3.00)
1 (19)	24.73 (23.63–26.14)	2.50 (–)
1 (49)	23.98 (23.26–25.34)	4.50 (3.25–4.75)
1 (99)	24.61 (23.92–25.69)	3.00 (2.25–5.00)

## Discussion

This study compared infection and dissemination of BTV-1w (GIB2007/1) within two colony-derived *Culicoides* species using two different means of virus quantification. Comparisons were drawn between C_q_ values generated from a front-line, diagnostic sqPCR used in reference laboratories across Europe, that measures the quantity of BTV RNA and an assay which was based on isolation and quantification of infectious virus. The results generated using the two assays were broadly similar and resembled those previously published that had examined BTV infection and dissemination in *C. sonorensis* and *C. nubeculosus*, but used alternative means of quantifying infectious virus to assess competence [21], [22], [32], [33]. Despite these similarities, observations were recorded that have significant relevance to the use of diagnostic assays for implication of field collected *Culicoides* as vectors and in the use of colony lines as models for BTV infection.

While the median quantity of infectious BTV detected in the original blood-meal taken by *C. sonorensis* was similar to previous studies, the range in titre (exceeding 2.5 log_10_ TCID_50_) was far greater [22], [34]. This variation was not reflected in the quantity of RNA directly extracted from homogenates and correlation between the two assays only improved once the original blood meal had been digested (by day 3 post-infection). A possible explanation for this discrepancy could lie in the fact that the homogenisation method used to disrupt samples prior to application to the KC-*C. sonorensis* cell line was not always sufficient to fully separate BTV bound to blood cells in the original blood meal. This would be likely to result in significant variation in the quantity of infectious BTV isolated and could in future be addressed by sonication of samples.

Following ingestion of a blood meal containing BTV, the titre of virus in *Culicoides* usually falls for a variable period of time, determined primarily by temperature [4], [21], [32]. During this ‘eclipse phase’, the number of *C. sonorensis* and *C. nubeculosus* containing infectious virus and BTV RNA fell, as virus imbibed with the blood meal was cleared by the majority of individuals. Evidence of replication of BTV was inferred where the quantity of BTV RNA in individuals exceeded that contained in the original blood meal. Previous studies had defined a threshold of 2.5–3.0 log_10_ TCID_50_ infectious BTV in *C. sonorensis* using detection systems based on isolation of virus from saliva followed by homogenisation and quantification of BTV from the whole insect [22], [28]. This value was very similar to the quantity of virus originally imbibed by *C. sonorensis*, as demonstrated by the fact that inferring maximum and minimum quantities of BTV RNA in fully disseminated individuals using either intrathoracic inoculation or membrane feeding in the current study did not provide significantly different estimates of competence when compared to those samples exceeding quantities of virus immediately following injection.

Values indicating replication of BTV to potentially transmissible levels and exceeding quantities contained in the original blood-meal were reached by 72 hours of incubation in *C. sonorensis* and within 120 hours of incubation in *C. nubeculosus* when measured either by sqPCR or isolation of infectious virus. This agreed closely with previous studies carried out using this *C. sonorensis* line, but different experimental procedures [22], [32]. Peak quantities of BTV RNA were reached by 7 days post-feeding in both species and in *C. sonorensis* were correlated with large quantities of infectious virus (≥4.5 log_10_ TCID_50_) comparable to those found in previous studies [22], [28]. The range of competence inferred from the mean C_q_ of BTV RNA in the original blood-meal for *C. sonorensis* (12–20% for days 7–11 post-infection) and *C. nubeculosus* (0–4%) again agreed closely with previous studies of these colony lines [21], [22], [35].

The findings of this study raise significant issues regarding the inference of competence in field collected *Culicoides* assessed purely by conventional PCR or rtRT-PCR where C_q_ values are not reported. In the case of the cohort of *C. sonorensis* from days 7–11, some 70% of individuals falling below the quantity of virus RNA in the original blood-meal would have had the potential to be reported as positive by these assays, but were very unlikely to have transmissible infections. It remains unclear at present what proportion of these individuals possessed persistent sub-transmissible infections or retained inactivated BTV RNA. It is clear, however, that the findings of previous studies from the sole use of these methods in *Culicoides* species are unreliable [15], [17], [36]–[38].

Comparison of the results from the current study with those where sqPCR C_q_ values have previously been reported also requires care. It has been demonstrated in this study that the results obtained from testing homogenised pools of varying numbers of uninfected *Culicoides*, each containing a single fully disseminated *C. sonorensis*, does not appear to influence the RNA levels detected. This is important as the prevalence of BTV-infected individuals in field-collected populations is almost universally low, reducing the likelihood of several infected individuals occurring in single pools [39]. Nevertheless, where large numbers of pools are used the presence of several infected individuals in a single pool cannot be entirely precluded although this can be partially addressed in statistical analysis [40].

Two published studies have been conducted to date that have reported C_q_ values from pools of field-collected *Culicoides* in the BTV-8 outbreak areas in Northern Europe. The first, carried out in Belgium and utilizing small pools of pigmented *Culicoides* (<10 individuals/pool) recorded a C_q_ range of 35.8–42.8 when utilising a cut-off value of 45 [19]. From the current study it appears likely that these ‘positive’ pools either contained no *Culicoides* with fully disseminated infections or that BTV RNA levels were reduced substantially during processing. In contrast, far higher RNA levels were reported from Germany, where seven pools of *Culicoides* recorded C_q_ values <25 when using a comparable assay to that used in the current study [18], despite using a sub-optimal extraction method [27]. The species of *Culicoides* present in the positive pools, however, remain uncertain as the relative brightness of bands from a conventional PCR assay was used for this purpose, a methodology that has not so far been successfully standardized for species identification. Due to this, the specific identity of *Culicoides* vector(s) involved in the single most costly incursion of BTV worldwide in recorded history remains unknown.

More recent studies with SBV have utilised an alternative approach that involves the decapitation of pigmented *Culicoides* collected in the field [41]. Pools of heads are processed for SBV RNA using sqPCR in an initial screen and then the positive individuals present in each pool are identified to species level using the remainder of the body of each *Culicoides*. In addition, a DNA sequence barcode based on a partial sequence of the mitochondrial cytochrome oxidase I gene is generated from positive individuals which enables the each implicated specimen to be identified to species level with at least some degree of certainty. This system echoes earlier studies that also processed individuals and used a multiplex-based assay for identification [42], but has the advantage of greater phylogenetic resolution. One likely limitation in take-up of this technique is that, anecdotally, SBV appears to be transmitted with far higher efficiency than BTV in northern European *Culicoides* and it is therefore more straightforward to define vector species through processing fewer individuals. Given the high-throughput nature of both the homogenisation technique and sqPCR assay used to detect arboviruses in *Culicoides*, however, and the absence of a requirement for a cold-chain to preserve infectious virus, it is likely that this technique has great potential to improve the reliability of work involving *Culicoides*-arbovirus relationships worldwide.

## References

[1] Carpenter S, Wilson A, Mellor PS (2009) *Culicoides* and the emergence of bluetongue virus in northern Europe. Trends Microbiol 17: 172–178. 19299131 10.1016/j.tim.2009.01.001

[2] OIE (2006) Bluetongue in the Netherlands. OIE Disease information 19: 34.

[3] Du Toit RM (1944) The transmission of blue-tongue and horsesickness by *Culicoides* . Onderstepoort J Vet 19: 7–16.

[4] Mellor PS (2000) Replication of arboviruses in insect vectors. J Comp Pathol 123: 231–247. 11041993 10.1053/jcpa.2000.0434

[5] Caracappa S, Torina A, Guercio A, Vitale F, Calabro A, et al (2003) Identification of a novel bluetongue virus vector species of *Culicoides* in Sicily. Vet Rec 153: 71–74. 12892265 10.1136/vr.153.3.71

[6] De Liberato C, Scavia G, Lorenzetti R, Scaramozzino P, Amaddeo D, et al (2005) Identification of *Culicoides obsoletus* (Diptera : Ceratopogonidae) as a vector of bluetongue virus in central Italy. Vet Rec 156: 301–304. 15786918 10.1136/vr.156.10.301

[7] Ferrari G, De Liberato C, Scavia G, Lorenzetti R, Zini M, et al (2005) Active circulation of bluetongue vaccine virus serotype-2 among unvaccinated cattle in central Italy. Preventive Vet Med-US 68: 103–113. 10.1016/j.prevetmed.2004.11.011 15820110

[8] Savini G, Goffredo M, Monaco F, Di Gennaro A, Cafiero MA, et al (2005) Bluetongue virus isolations from midges belonging to the *Obsoletus* complex (*Culicoides*, Diptera : Ceratopogonidae) in Italy. Vet Rec 157: 133–139. 16055660 10.1136/vr.157.5.133

[9] Mellor PS, Pitzolis G (1979) Observations on breeding sites and light-trap collections of *Culicoides* during an outbreak of bluetongue in Cyprus. Bull Entomol Res 69: 229-&.

[10] Purse BV, Nedelchev N, Georgiev G, Veleva E, Boorman J, et al (2006) Spatial and temporal distribution of bluetongue and its *Culicoides* vectors in Bulgaria. Med Vet Entomol 20: 335–344. 17044886 10.1111/j.1365-2915.2006.00636.x

[11] Torina A, Caracappa S, Mellor PS, Baylis M, Purse BV (2004) Spatial distribution of bluetongue virus and its *Culicoides* vectors in Sicily. Med Vet Entomol 18: 81–89. 15189232 10.1111/j.0269-283X.2004.00493.x

[12] Mellor PS, Wittmann EJ (2002) Bluetongue virus in the Mediterranean Basin 1998–2001. Vet J 164: 20–37. 12359482 10.1053/tvjl.2002.0713

[13] Jennings DM, Mellor PS (1988) The vector potential of British *Culicoides* species for bluetongue virus. Vet Microbiol 17: 1–10. 2845631 10.1016/0378-1135(88)90074-0

[14] Carpenter S, Lunt HL, Arav D, Venter GJ, Mellor PS (2006) Oral susceptibility to bluetongue virus of *Culicoides* (Diptera : Ceratopogonidae) from the United Kingdom. J Med Entomol 43: 73–78. 16506450 10.1093/jmedent/43.1.73

[15] Meiswinkel R, van Rijn P, Leijs P, Goffredo M (2007) Potential new *Culicoides* vector of bluetongue virus in northern Europe. Vet Rec 161: 564–565. 17951565 10.1136/vr.161.16.564

[16] Dijkstra E, van der Ven IJK, Melswinkel R, Holzel DR, van Rijn PA, et al (2008) *Culicoides chiopterus* as a potential vector of bluetongue virus in Europe. Vet Rec 162: 424–424. 10.1136/vr.162.13.422-a 18375991

[17] Mehlhorn H, Walldorf V, Klimpel S, Jahn B, Jaeger F, et al (2007) First occurrence of *Culicoides obsoletus*-transmitted Bluetongue virus epidemic in Central Europe. Parasitol Res 101: 219–228. 17385085 10.1007/s00436-007-0519-6

[18] Hoffmann B, Bauer B, Bauer C, Batza HJ, Beer M, et al (2009) Monitoring of Putative Vectors of Bluetongue Virus Serotype 8, Germany. Emerg Infect Dis 15: 1481–1484. 19788820 10.3201/eid1509.090562 PMC2819873

[19] Vanbinst T, Vandenbussche F, Vandemeulebroucke E, De Leeuw I, Deblauwe I, et al (2009) Bluetongue Virus Detection by Real-Time RT-PCR in *Culicoides* Captured During the 2006 Epizootic in Belgium and Development of an Internal Control. Transbound Emerg Dis 56: 170–177. 19432638 10.1111/j.1865-1682.2009.01077.x

[20] Bowne JG, Jones RH (1966) Observations on bluetongue virus in the salivary glands of an insect vector, *Culicoides variipennis* . Virology 30: 127–133. 5911888 10.1016/s0042-6822(66)81016-4

[21] Wittmann EJ, Mellor PS, Baylis M (2002) Effect of temperature on the transmission of orbiviruses by the biting midge, *Culicoides sonorensis* . Med Vet Entomol 16: 147–156. 12109708 10.1046/j.1365-2915.2002.00357.x

[22] Fu H, Leake CJ, Mertens PPC, Mellor PS (1999) The barriers to bluetongue virus infection, dissemination and transmission in the vector, *Culicoides variipennis* (Diptera : Ceratopogonidae). Arch Virol 144: 747–761. 10365165 10.1007/s007050050540

[23] Hoffmann B, Scheuch M, Hoper D, Jungblut R, Holsteg M, et al (2012) Novel Orthobunyavirus in Cattle, Europe, 2011. Emerg Infect Dis 18: 469–472. 22376991 10.3201/eid1803.111905 PMC3309600

[24] Jones RH (1964) Mass production methods in rearing *Culicoides variipennis* (Coquillett). Bull World Health Organ 31: 571–572. 14272470 PMC2555016

[25] Boorman J (1974) Maintenance of laboratory colonies of *Culicoides variipennis* (Coq), *Culicoides nubeculosus* (Mg) and *Culicoides riethi* Kieffer (Diptera, Ceratopogonidae). Bull Ent Res 64: 371–377.

[26] Wechsler SJ, McHolland LE, Tabachnick WJ (1989) Cell-lines from *Culicoides variipennis* (Diptera, Ceratopogonidae) support replication of bluetongue virus. J Invert Pathol 54: 385–393. 10.1016/0022-2011(89)90123-7 2553822

[27] Veronesi E, Mertens PPC, Shaw AE, Brownlie J, Mellor PS, et al (2008) Quantifying bluetongue virus in adult *Culicoides* biting midges (Diptera : Ceratopogonidae). J Med Ent 45: 129–132. 10.1603/0022-2585(2008)45[129:qbviac]2.0.co;2 18283953

[28] Jennings DM, Mellor PS (1987) Variation in the responses of *Culicoides variipennis* (Diptera, Ceratopogonidae) to oral infection with bluetongue virus. Arch Virol 95: 177–182. 3038052 10.1007/BF01310778

[29] Shaw AE, Monaghan P, Alpar HO, Anthony S, Darpel KE, et al (2007) Development and initial evaluation of a real-time RT-PCR assay to detect bluetongue virus genome segment 1. J Virol Methods 145: 115–126. 17586061 10.1016/j.jviromet.2007.05.014

[30] Mecham JO (2006) Detection and titration of bluetongue virus in *Culicoides* insect cell culture by an antigen-capture enzyme-linked immunosorbent assay. J Virol Methods 135: 269–271. 16672164 10.1016/j.jviromet.2006.03.002

[31] Finnay DJ (1964) Statistical Methods in Biological Assay. London: Griffin.

[32] Mullens BA, Tabachnick WJ, Holbrook FR, Thompson LH (1995) Effects of temperature on virogenesis of bluetongue virus serotype 11 in *Culicoides variipennis sonorensis* . Med Vet Entomol 9: 71–76. 7696691 10.1111/j.1365-2915.1995.tb00119.x

[33] Foster NM, Jones RH (1979) Multiplication rate of bluetongue virus in the vector *Culicoides variipennis* (Diptera: Ceratopogonidae) infected orally. J Med Entomol 15: 302–303. 220423 10.1093/jmedent/15.3.302

[34] Jennings DM, Mellor PS (1987) Variation in the responses of *Culicoides variipennis* (Diptera, Ceratopogonidae) to oral infection with bluetongue virus. Arch Virol 95: 177–182. 3038052 10.1007/BF01310778

[35] Carpenter S, Wilson A, Barber J, Veronesi E, Mellor P, et al. (2011) Temperature Dependence of the Extrinsic Incubation Period of Orbiviruses in *Culicoides* Biting Midges. Plos One 6. 10.1371/journal.pone.0027987 PMC3220716 22125649

[36] Dijkstra E, van der Ven, I.J.K. and Meiswinkel (2008) R (2008) *Culicoides chiopterus* as a potential vector of bluetongue virus in Europe. Vet Rec 162: 422. 10.1136/vr.162.13.422-a 18375991

[37] Becker ME, Reeves WK, Dejean SK, Emery MP, Ostlund EN, et al (2010) Detection of Bluetongue Virus RNA in Field-Collected *Culicoides* spp. (Diptera: Ceratopogonidae) Following the Discovery of Bluetongue Virus Serotype 1 in White-Tailed Deer and Cattle in Louisiana. J Med Entomol 47: 269–273. 20380309 10.1603/me09211

[38] Sabio IJ, Mackay AJ, Roy A, Foil LD (2006) Detection of West Nile virus RNA in pools of three species of ceratopogonids (Diptera : Ceratopogonidae) collected in Louisiana. J Med Entomol 43: 1020–1022. 17017242 10.1603/0022-2585(2006)43[1020:downvr]2.0.co;2

[39] Mellor PS, Boorman J, Baylis M (2000) *Culicoides* biting midges: Their role as arbovirus vectors. Ann Rev Entomol 45: 307–340. 10761580 10.1146/annurev.ento.45.1.307

[40] Gu WD, Unnasch TR, Katholi CR, Lampman R, Novak RJ (2008) Fundamental issues in mosquito surveillance for arboviral transmission. T R Soc Trop Med H 102: 817–822. 10.1016/j.trstmh.2008.03.019 PMC2577025 18466940

[41] Elbers ARW, Meiswinkel R, van Weezep E, van Oldruitenborgh-Oosterbaan MMS, Kooi EA (2013) Schmallenberg virus in *Culicoides* spp. biting midges, the Netherlands, 2011. Emerg infect dis 19: 106–109. 23260040 10.3201/eid1901.121054 PMC3558002

[42] Carpenter S, McArthur C, Selby R, Ward R, Nolan DV, et al (2008) Experimental infection studies of UK *Culicoides* species midges with bluetongue virus serotypes 8 and 9. Vet Rec 163: 589–592. 19011244 10.1136/vr.163.20.589

